# Supplementary material for: Learning curve and surgical outcome of robotic assisted colorectal surgery with ERAS program
Source: Sci Rep. 2022 Nov 29;12:20566. doi: 10.1038/s41598-022-24665-w (PMC9709162; doi:10.1038/s41598-022-24665-w)
Supplement: Supplementary file 2 — Supplementary Information 2. [file 41598_2022_24665_MOESM2_ESM.zip › RAL Raw data-Ñ[▒K/116/Patho_ñw╝╨░O▒Kñσ.pdf]

臺中榮民總醫院 臺中市臺灣大道四段1650號 病理部活體組織切片 臨床醫學實驗及病理診斷中心 檢驗(查)報告

[redacted]

Pathologic diagnosis:
Ascending colon, terminal ileum, and appendix, robot assisted right hemicolectomy --- Adenocarcinoma in situ, arising in a tubulovillous adenoma, of ileocecal valve. The appendix and bilateral cut ends and mesocolic lymph nodes (0/41) are free of tumor.

Ancillary study for diagnosis:
Deep cut done for section G.

Prognostic and predictive factor:
1. Tumor size: 0.6 cm.
2. Depth of invasion: No invasion (high-grade dysplasia).
3. Histologic grade: Not applicable.
4. Angiolymphatic invasion: Not identified.
5. Perineural invasion: Not identified.
6. Tumor deposits (discontinuous extramural extension): Not identified.
7. Macroscopic tumor perforation: Not identified.
8. Surgical Margins: a) Proximal Margin: Uninvolved by tumor, distance of tumor from margin: 7 cm. b) Distal Margin: Uninvolved by tumor, distance of tumor from margin: 12 cm. c) Radial or Mesenteric Margin: Uninvolved by tumor, distance of tumor from margin: 5 cm.
9. Lymph node status: Uninvolved (0/41).
10. Extranodal involvement: Not identified.
11. Tumor regression grading S/P CCRT: No known presurgical therapy.
12. Type of polyp in which invasive carcinoma arose: Tubulovillous adenoma.
13. Additional pathologic findings: Not identified.
14. Pathological TNM Stage: pTisN0 (According to the eighth edition, American Joint Committee on Cancer Staging Guidelines for Tumors).
15. TNM descriptors: Not applicable.

Gross description:
The specimen consists of a segment of colon, 12 cm in length, and 7 cm in proximal circumference, 6 cm in distal cut end circumference, and a segment of terminal ileum, 7 cm in length, and 4 cm in cut end circumference. An unremarkable appendix, 7 cm, is noted. A tan soft to firm bulging tumor, 4x2x1 cm, is noted on the ileocecal valve. On cross section, the tumor confined to mucosa. The appendix and both cut ends are free of tumor. Representative part for section: A) proximal cut end B) distal cut end C) appendix D) circumferential margin E-H) tumor J-N) lymph nodes.

Note:
This case has been peer reviewed by two doctors.
#T-58650_2 #M-81402_2 9 1105 U0000F
#T-C4560_2 #M-09183_2 0 1105 000000
#T-59200_2 #M-09183_2 0 1105 000000

已電子簽章

敬告：本單僅供醫師參考使用，不具任何申請效力！！
請病患妥為保管，若不慎遺失需自負個人資料外洩責任！
